# Connecting Alzheimer’s Disease With Diabetes Mellitus Through Amyloidogenic Evolvability

**DOI:** 10.3389/fnagi.2020.576192

**Published:** 2020-10-28

**Authors:** Gilbert Ho, Yoshiki Takamatsu, Ryoko Wada, Shuei Sugama, Masaaki Waragai, Takato Takenouchi, Eliezer Masliah, Makoto Hashimoto

**Affiliations:** ^1^PCND Neuroscience Research Institute, Poway, CA, United States; ^2^Tokyo Metropolitan Institute of Medical Science, Tokyo, Japan; ^3^Department of Physiology, Nippon Medical School, Tokyo, Japan; ^4^Institute of Agrobiological Sciences, National Agriculture and Food Research Organization, Tsukuba, Japan; ^5^Division of Neurosciences, National Institute on Aging, National Institutes of Health, Bethesda, MD, United States

**Keywords:** Alzheimer’s disease, diabetes mellitus, evolvability, antagonistic pleiotropy, adiponectin paradox

## Abstract

Type 2 diabetes mellitus (T2DM) has been clearlylinked to oxidative stress and amylin amyloidosis in pancreatic β-cells. Yet despite extensive investigation, the biological significance of this is not fully understood. Recently, we proposed that Alzheimer’s disease (AD)-relevant amyloidogenic proteins (APs), such as amyloid-β (Aβ) and tau, might be involved in evolvability against diverse stressors in the brain. Given the analogous cellular stress environments shared by both T2DM and AD, the objective of this study is to explore T2DM pathogenesis from the viewpoint of amyloidogenic evolvability. Similar to AD-related APs, protofibrillar amylin might confer resistance against the multiple stressors in β-cells and be transmitted to offspring to deliver stress information, in the absence of which, type 1 DM (T1DM) in offspring might develop. On the contrary, T2DM may be manifested through an antagonistic pleiotropy mechanism during parental aging. Such evolvability-associated processes might be affected by parental diabetic conditions, including T1DM and T2DM. Furthermore, the T2DM-mediated increase in AD risk during aging might be attributed to an interaction of amylin with AD-related APs through evolvability, in which amylin protofibrillar formation presumably caused by adiponectin (APN) resistance could increase protofibril formation of AD-related APs in evolvability and subsequently lead to T2DM promotion of AD through antagonistic pleiotropy in aging. This suggests that targeting APN combined with an anti-T2DM agent might be therapeutic against neurodegeneration. Collectively, T1DM and T2DM might be linked through amylin evolvability, and a better understanding of amyloidogenic evolvability might also reveal clues to therapeutic interventions for AD comorbid with T2DM.

## Introduction

Accumulating evidence suggests that type 2 diabetes mellitus (T2DM) may drive the pathogenesis of various nervous system disorders, including ischemia, depression, and neurodegenerative conditions (Atlantis et al., 2014; Takamatsu et al., 2017). In Alzheimer’s disease (AD), T2DM may increase the risk of mild cognitive impairment and subsequent progression to dementia (Watts et al., 2013; Ng et al., 2016). Additionally, T2DM has been linked to other neurodegenerative disorders, including Parkinson’s disease (PD) and Huntington’s disease (HD; Aviles-Olmos et al., 2013; Montojo et al., 2017). Yet despite a plethora of such observations, the precise mechanistic underpinning of the comorbidity between T2DM and neurodegeneration remains elusive.

Our recent work suggests that the evolvability of amyloidogenic proteins (APs) relevant to neurodegeneration, such as β-amyloid (Aβ) in AD, α-synuclein (αS) in PD, and huntingtin in HD, might be physiologically important (Hashimoto et al., 2018a,b,c; Takamatsu et al., 2018). More specifically, APs might act as vehicles to transgenerationally deliver information regarding diverse biological stressors to cope with such forthcoming stressors in offspring’s brain (Hashimoto et al., 2018a). On the contrary, evolvability might also cause aging-associated neurodegenerative disease through the antagonistic pleiotropy mechanism during the course of parental aging (Hashimoto et al., 2018b). Since evolvability is critical for reproduction, neurodegenerative diseases in aging have evaded “weeding out” by natural selection during evolution.

Given analogous pathology between T2DM and neurodegenerative disorders in terms of amyloidosis-associated stressors (Abedini and Schmidt, 2013; Singh et al., 2015), common mechanisms might form the basis for both groups of disorders. In this context, the main objective of this article is to discuss the mechanisms by which T2DM might increase AD risk from the viewpoint of amyloidogenic evolvability. We hypothesize that amylin protofibrils might confer resistance against multiple stressors in β-cells in the pancreas, which might be transgenerationally transmitted *via* the germ line to offspring, the absence of which might lead to type 1 DM (T1DM), and where T2DM in parents might promote amylin evolvability, while T1DM in parents might increase T1DM in offspring. Furthermore, we assume that the comorbidity of AD and T2DM in aging might be attributed to cross seeding of APs, which may stimulate evolvability. Yet the increased risk of AD from T2DM during aging might be due to upstream amylin activity relative to other more prominent APs, such as Aβ and tau, in evolvability, where the adiponectin (APN) paradox might be important. Collectively, DM and AD might be connected through amyloid evolvability, which might shed light on novel therapeutic strategies against such comorbid disorders.

## Evolvability of Amyloidogenic Proteins Relevant to Type 2 Diabetes Mellitus

Since T2DM is associated with increased biological stressors and formation of amyloid-like fibrils of amylin in pancreatic β cells, we propose that amylin evolvability might be involved in the pathogenesis of T2DM.

### Amylin and Insulin Are Amyloidogenic Proteins

Amylin, also called islet amyloid polypeptide, is a 37-residue peptide that is co-secreted with insulin by pancreatic β-cells (Mietlicki-Baase, 2016). The normal functions of amylin include inhibition of glucagon secretion and reduction of the gastric emptying rate, effects that contribute to the maintenance of postprandial glucose homeostasis (Mietlicki-Baase, 2016). Amylin is prone to aggregate, leading to the formation of amyloid fibrils with characteristic β-sheet structures (Jaikaran and Clark, 2001). Consistent with this, amylin is the major component of the islet amyloid found in the pancreas in T2DM, suggesting that amylin may play a significant role in the pathogenesis of T2DM (Luca et al., 2007). In addition, insulin, a 51-residue protein that is composed of a dimer of an A- and a B-chain linked by disulfide bonds (Brange and Langkjoer, 1993), also forms amyloid fibrils. Insulin, and its related peptide, insulin-like growth factor-1 (IGF-1), are essential to multiple physiological processes, including cell proliferation, differentiation, and survival, in addition to the regulation of energy storage and glucose metabolism (Stewart and Rotwein, 1996; Dadon et al., 2012). In contrast to amylin, the biological role of insulin fibril formation remains unclear. Therefore, our article will henceforth refer exclusively to amylin in this context.

### Evolvability of Amylin

**Figure 1:**
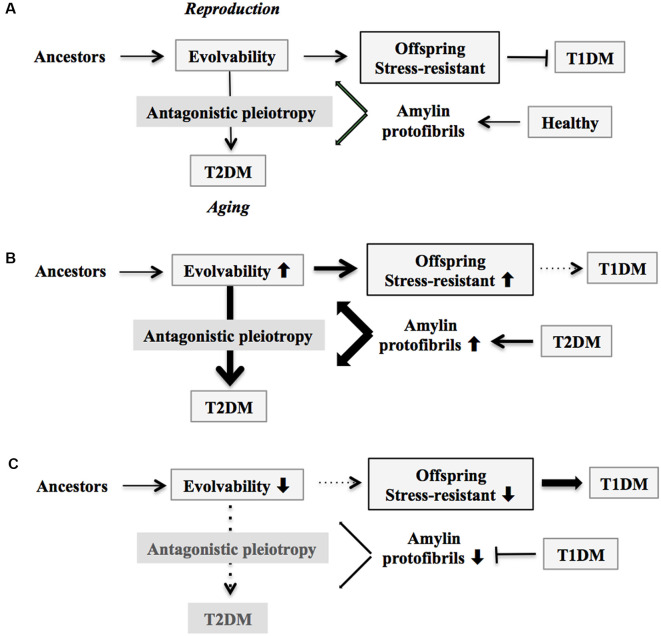
Schematic of disease manifestation caused by alterations in amylin evolvability. **(A)** Amylin protofibrils might confer resistance against multiple stressors in parental β-cells in the pancreas under the healthy conditions and are transgenerationally transmitted to offspring during reproduction to deliver the stress information. By virtue of this, the β-cells in the pancreas in offspring can cope with forthcoming multiple stressors in β-cells in the pancreas. Thus, amylin evolvability is an epigenetic phenomenon that is beneficial in evolution. However, amylin protofibrils might lead to type 2 diabetes mellitus (T2DM) during parental aging through the antagonistic pleiotropy mechanism. **(B)** Increased evolvability of amylin protofibrils by various causes, such as T2DM (thick bold line), may result in an efficient delivery of information about stressors for offspring, leading to reduced frequency of T1DM in offspring (thin dot line) and increased frequency of T2DM in parents (thick bold line). **(C)** In contrast, inefficient delivery of the information of stresses due to decrease of amylin evolvability caused by type 1 diabetes mellitus (T1DM) in parents may result in the increased frequency of T1DM in offspring (thick bold line) and the decreased frequency of T2DM in parents (thin dot line).

Because APs such as amylin are intrinsically disordered proteins composed of heterogeneous structures (Moore et al., 2018), APs might be involved in resistance against diverse stressors (Takamatsu et al., 2018). In addition, amylin toxicity in pancreatic β-cells has been well described in the pathogenesis of T2DM (Bharadwaj et al., 2017). Collectively, this may be comparable with the hormesis conferred by amyloidogenic evolvability (Hashimoto et al., 2018a). Because evolvability is defined as the capacity of a population of organisms to generate not only genetic diversity, but also adaptive genetic diversity, often overriding natural selection (Kirschner and Gerhart, 1998), both hormesis and heredity are critical steps in evolvability. In humans, AP protofibrils that encode information regarding biological and environmental stressors might be transmitted to offspring *via* germ lines (Hashimoto et al., 2018a). Although monomers of APs are unstable due to their intrinsically disordered nature (Takamatsu et al., 2018), formation of protofibrils might confer greater stability, which might provide an advantage for the transgenerational transmission of APs to offspring (Takamatsu et al., 2018). The presence of circulatory amylin is consistent with the transgenerational transmission *via* germ cells, although little is known about the expression of amylin in gonadal tissues. Yet given that amyloid fibrils are abundantly expressed in the semen (Roan et al., 2017), further exploration of this is definitely warranted. Taken together, our concept proposes that amylin protofibrils may convey stress information from parental pancreatic β-cells to an offspring’s pancreas. By virtue of amylin evolvability, offspring can then better cope with the forthcoming stressors, reducing the risk of developing T1DM (Figure 1A). Indeed, it was previously shown that amylin-knockout mice developed a more severe form of alloxan-induced diabetes (Mulder et al., 2000), which is consistent with our hypothesis that amylin protofibrils might be involved in evolvability of β-cells in the pancreas.

On the other hand, T2DM might manifest in later life through the mechanism of antagonistic pleiotropy during parental aging (Figure 1A). Briefly, according to the antagonistic pleiotropy hypothesis, a prominent theory of aging proposed by G. C. Williams a half-century ago, certain genes whose functions are beneficial during reproductive stages may in turn exert adverse effects later in aging (Williams, 1957). Such a view may explain why T2DM, a primarily post-reproductive and biologically disadvantageous disorder, has emerged and persisted across evolution. Yet it is recognized that T2DM is a complex disorder further modulated by interaction of a combination of susceptible genes and perhaps lifestyle factors such as exercise and diet.

## Connecting Type 1 Diabetes Mellitus and Type 2 Diabetes Mellitus Through Amyloidogenic Evolvability

Although it is generally believed that T1DM occurs during youth and may be etiologically distinct from T2DM, which emerges during later adulthood, we alternatively propose that the two types of DM might actually be closely linked through amyloidogenic evolvability.

### The Conventional View of Type 1 Diabetes Mellitus and Type 2 Diabetes Mellitus Pathogenesis

In T1DM, which accounts for 5–10% of all diabetes cases and occurs in approximately 0.3% of young individuals, amassed evidence suggests that various etiologies, such as autoimmune dysfunction and viral infection, might be pathogenetically involved (Daneman, 2006; Menke et al., 2013). Furthermore, recent genome-wide association studies have shown that the major susceptibility for the T1DM locus maps to the HLA class II genes at 6p21, which accounts for up to 30~50% of genetic risk of T1DM, with minor contributions to disease risk from several other non-MHC loci (Steck and Rewers, 2011). On the contrary, the more common T2DM, estimated to be ~7% of the general population, in contrast to early-onset T1DM, most often begins in those over the age of 65 years (although some early-onset T2DM cases are reported; Deshpande et al., 2008). Despite similar pathology in terms of dysfunctional pancreatic β cells, T1DM and T2DM are understood to be etiologically different entities.

### Equilibrium Between Type 1 Diabetes Mellitus in Offspring and Parental Type 2 Diabetes Mellitus Through Amylin Evolvability

Based on our evolvability concept, stress information from parental/adult pancreatic β-cells could be delivered to their offspring through amylin protofibrils. The resulting pancreatic β-cells in offspring should therefore become more resistant against such stressors (Figure 1A).

According to our view, the greater the stress information from parental pancreatic β-cells that is delivered through amylin protofibrils to offspring through increased evolvability, the more resistant the pancreatic β-cells in offspring become against stressors, and the less prone they are to developing T1DM. This could result from various factors, including amylin missense mutation (Akter et al., 2016), T2DM in both parents, and maternal gestational diabetes mellitus (GDM). Consistent with our view, it was previously shown that parental history of T2DM is associated with a later onset of T1DM, the metabolic syndrome, and a metabolic profile related to insulin resistance (Thorn et al., 2009). Because of the action of antagonistic pleiotropy, T2DM is expressed more prominently during aging (Figure 1B).

Yet if the parents are afflicted with T1DM, then reduced evolvability associated with the down-regulation of amylin protofibrils might result in the delivery of less stress information to offspring, and as a consequence, T1DM risk may dramatically increase in offspring (Figure 1C). Supporting this, T1DM is preferentially transmitted from parents to offspring, although the mechanism of gender difference is obscure (Guo and Tuomilehto, 2002). Of interest, the inverse relationship between T1DM in offspring and parental T2DM is reminiscent of the concept of transgenerational equilibrium previously described between parental AD and schizophrenia in offspring related to amyloid evolvability (Takamatsu et al., 2019), raising a possibility that certain chronic disorders in offspring and their aging-associated amyloidogenic disorder counterparts in parents may exist in an inverse relationship through amyloidogenic evolvability.

### Are There any Roles for Gestational Diabetes Mellitus in Amylin Evolvability?

The third diabetic subtype, GDM, occurs among pregnant women without a previous history of DM (Kampmann et al., 2015). GDM, with a prevalence of ~39% of pregnancies, correlates with increased maternal obesity (Carpenter, 2007; Egan et al., 2017). GDM shows a complex etiology, with genetic and environmental factors implicated across mechanistic and epidemiological studies. Compared with T1DM and T2DM, there have been fewer studies on the etiological basis of GDM, because of its transient nature and resolution upon delivery of the infant. Thus, a possible etiologic role for GDM might also relate to evolvability. Given that GDM is associated with increased insulin resistance (Catalano, 2014), GDM might be involved in transmitting amylin protofibrils encoding stress information from parental pancreatic β-cells to offspring in order to mitigate the risk of T1DM in offspring. On the other hand, symptomatic T2DM might emerge later during parental aging. If this notion can be verified, GDM may become central to linking T1DM in offspring and parental T2DM through amylin evolvability. Yet, presently, there is no evidence that amylin can cross the transplacental barrier, which is generally believed to be permeable to only small molecules (Berveiller et al., 2016). Therefore, it would be fair to conclude that GDM might be unlikely to promote amylin evolvability. As will be described later for T2DM, GDM might represent an antagonistic pleiotropy of amylin evolvability. Consistently, after the occurrence of GDM, there is a higher likelihood of developing subsequent maternal T2DM, as well as possible abnormal cardiometabolic phenotypes in offspring (Carpenter, 2007; Kawasaki et al., 2018).

Moreover, pregnant women often experience memory dysfunction through the course of their pregnancy (John et al., 2018), which is generally attributed to elevated hormone levels affecting the brain, although the precise mechanism is unknown. Similar to GDM, gestational dementia might be relevant to amyloid evolvability, perhaps involving Aβ and tau. To the best of our knowledge, limited information is currently available regarding changes in brain APs during pregnancy.

## Comorbidity of Alzheimer’s Disease and Type 2 Diabetes Mellitus

Supposing that both T2DM and AD are antagonistic phenomena derived from amyloidogenic evolvability during reproduction, it follows that comorbidity of T2DM with AD in aging might be attributed to the synergistic interaction of amylin with APs, including Aβ and tau, in regulating evolvability during reproduction.

### The Conventional View of the Comorbidity of Alzheimer’s Disease and Type 2 Diabetes Mellitus

With regard to the mechanisms underlying increased neurodegeneration related to T2DM, numerous studies have shown that both T2DM and AD are associated with various common pathological features, including impaired insulin resistance, endoplasmic reticulum stress, oxidative stress, protein aggregation, inflammation and altered gene expression (Vannuvel et al., 2013; Hokama et al., 2014; Singhal et al., 2014; Rosales-Corral et al., 2015). Furthermore, it was described that vascular dysfunction caused by T2DM might be relevant to AD (Wang et al., 2014). These results, however, were pathological observations, and do not necessarily explain the rationale for the emergence of these two comorbid aging-related disorders in evolution.

### Comorbidity of Alzheimer’s Disease and Type 2 Diabetes Mellitus From the Viewpoint of Evolvability

**Figure 2:**
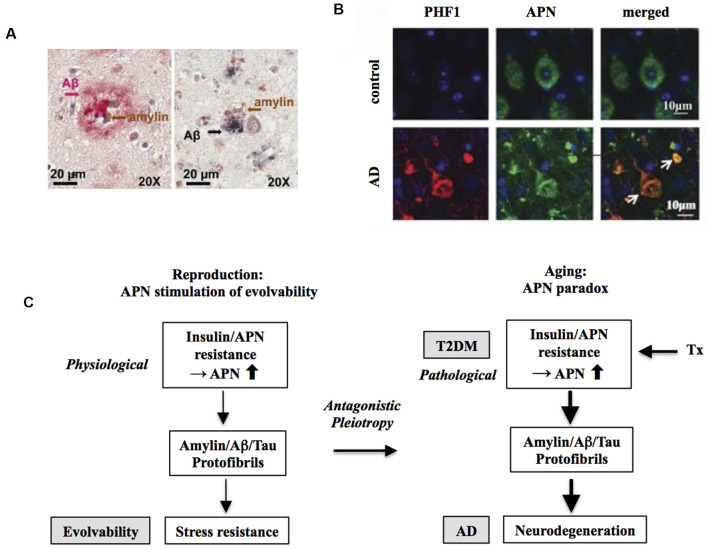
Increased risk of Alzheimer’s disease (AD) by T2DM as an antagonistic pleiotropy of evolvability. **(A)** Co-localization of amylin and amyloid-β (Aβ) is shown in brain parenchyma of AD patients with anti-amylin and anti-Aβ antibodies. The left panel shows a brain section from an AD patient without clinically demonstrated T2DM, while the right panel is derived from a diabetic patient with AD. Clusters of small amylin plaques adjacent to, or surrounded by larger Aβ deposits, are present. Modified from Jackson et al. (2013) with permission. **(B)** Adiponectin (APN)-positive neurofibrillary tangles (NFTs) in AD brain. Confocal laser scanning microscopy of double immunofluorescence of control and AD brains using anti-tau antibody PHF (lower) and anti-APN antibody showed that APN co-localized with tau in AD. White arrows indicate NFTs identified by the presence of PHF-1 immunoreactive abnormal fibrous inclusions, which were within the perikaryal cytoplasm of neurons. Scale bar = 10 μm. Modified from Waragai et al. (2016) with permission. **(C)** Schematic diagram of the mechanism underlying the APN paradox in T2DM-mediated increased AD risk. Presumably, amylin protofibrillar formation caused by APN resistance could up-regulate AP protofibrils in evolvability, which might manifest as T2DM stimulation of AD as the antagonistic pleiotropy in aging.

In this regard, evolvability might then underlie these biological phenomena. Provided that both T2DM and AD might be antagonistic phenomena derived from evolvability, it follows that amylin and amyloid-β (Aβ) and/or tau might interact cooperatively rather than function independently. Given the prevailing concept of cross-seeding (CS) of APs (Morales et al., 2013), perhaps the CS of amylin and Aβ and/or tau may be more potent than either monomer to stimulate various aspects of evolvability, such as hormesis and transgenerational transmission. Yet CS of these APs may manifest later in life as comorbid T2DM and AD through antagonistic pleiotropy in parental aging. Indeed, amylin interacts with Aβ and tau in both the pancreas and hippocampus in the brain of diabetic patients with AD (Figure 2A; Jackson et al., 2013).

### Importance of Common Modifiers of Alzheimer’s Disease and Type 2 Diabetes Mellitus From the Viewpoint of Evolvability

Notably, abnormally high levels of D-ribose have been observed in both T1DM (Yu et al., 2019) and T2DM (Su and He, 2014). Since administration of D-ribose induces the yield of Aβ and hyper-phosphorylated tau in the brain (Wu et al., 2015, 2019; Li et al., 2020), D-ribose might be also important for induction of AD. Thus, D-ribose might affect evolvability of amylin and Aβ, the dysmetabolism of which might lead to manifestation as comorbidity of AD and T2DM in aging. Indeed, it is possible that there might be many factors/stressors that may be attributed to the comorbidity of AD and T2DM. These include formaldehyde and amylin glycation.

### Comorbidity of Alzheimer’s Disease With Other Metabolic Disorders

Considering that AD is frequently comorbid with many other types of aging-associated diseases, similar mechanisms might be applied to the comorbidity of AD with these other conditions. Indeed, an increasing prevalence of associated metabolic disorders, including obesity, hypertension, hyperlipidemia, and atherosclerosis, is associated with an expanding adult and elderly population worldwide (Takamatsu et al., 2017). One may argue, however, that metabolic disorders other than T2DM are not always associated with amyloid fibril formation yet frequently promote neurodegeneration (Pugazhenthi, 2017). In this regard, evolvability of APs, including Aβ and tau, might be increased through mechanisms other than aggregative protein–protein interactions. For instance, cholesterol, which may play a central role in dyslipidemia, might stimulate misfolding and aggregation of APs in evolvability as well as in neurodegeneration and T2DM (Singh et al., 2015). Also, catecholamines possibly related to essential hypertension might be relevant (Goldstein, 1983). Aβ was shown to undergo regulated co-secretion with neuropeptide and catecholamine neurotransmitters (Toneff et al., 2013), and catecholamines were also shown to stimulate protein deposition in AD and PD (Bharath and Andersen, 2004). Thus, catecholamines might be involved in evolvability, which may manifest as essential hypertension as an antagonistic pleiotropy relationship in aging. Collectively, it is possible that the comorbidity of AD with metabolic disorders might represent antagonistic pleiotropy attributed to increased evolvability due to the CS of APs and other factors.

## Role of Adiponectin Paradox Linking Type 2 Diabetes Mellitus and Alzheimer’s Disease

The concept of the CS of APs suggests that the pathogenesis of T2DM might be equivalent to that in AD but fails to explain how T2DM could be upstream of AD. Given that AD risk might be increased by T2DM, perhaps the mechanistic action of amylin in evolvability may be situated upstream to APs, including Aβ and tau.

In this regard, we hypothesize that APN might be involved. APN is a multifunctional adipokine that is important in insulin receptor signaling sensitization and an anti-inflammatory role (Waragai et al., 2017). Although APN is generally protective, it has been well characterized that APN might be critically involved in promoting aging-associated chronic diseases, such as chronic heart failure and chronic kidney disease, the so-called APN paradox (Menon et al., 2006; Kizer, 2014). Notably, a recent study suggests that the APN paradox is also observed in the nervous system (Waragai et al., 2020). APN is neuroprotective and mitigates neurodegeneration in both cellular and animal experimental systems. Nonetheless, in cohort studies, it was shown that hyperadiponectinemia correlates well with neuropathological features, such as amyloidosis and cognitive deficits, suggesting that APN paradox might be involved in AD pathogenesis (Wennberg et al., 2016; Waragai et al., 2020). Consistent with this notion, it was shown that APN associated with phospho-tau in the AD brain, suggesting that APN might be involved in the tangle formation (Figure 2B).

Given that the APN paradox might be critical in promoting AD (Waragai et al., 2020), it is possible that the APN paradox in aging might be an antagonistic pleiotropy phenomenon derived from evolvability during reproduction (Waragai et al., 2020). In this context, APN resistance/insulin resistance might be a physiologically caused by fibrillar amylin in evolvability, which might later be manifest as T2DM stimulation of AD (Figure 2C). Consistently, insulin resistance was previously shown in cavefish as an adaptation to a nutrient-limited environment (Riddle et al., 2018), indicating that insulin resistance can be beneficially activated under the physiological conditions. Notably, it was recently described that large-scale proteomic analysis of AD brain and cerebrospinal fluid revealed early changes in energy metabolism associated with glial activation (Johnson et al., 2020), which is consistent with involvement of the APN paradox in the pathogenesis of AD (Waragai et al., 2020).

Similar to AD, a recent prospective cohort study showed that higher serum APN concentrations were observed in incident cancers and are associated with cancer-related deaths in T2DM, suggesting that the APN paradox might be significant in cancer related to T2DM (Lee et al., 2020).

## Implications for Therapeutic Interventions

In view of the possible involvement of the APN paradox in linking neurodegeneration to metabolic disease, this may account for the pathological positioning of T2DM upstream of AD in aging, suggesting that therapeutic targeting of APN might be a viable strategy.

### Targeting Adiponectin Expression

For this purpose, suppressing APN expression by various methods, such as antisense oligonucleotides against APN mRNA (Rinaldi and Wood, 2018) and immunotherapy against the APN protein (Lannfelt et al., 2014), might effectively decrease formation of APs protofibrils, leading to suppression of APN paradox and AD (Figure 2C). However, the majority of the previous studies describe that the potential of APN activity for therapeutic purposes based on the presumption that APN is protective. Therefore, it is necessary to bear in mind the possibility of a therapeutic “trade-off” by suppression of APN expression.

Notably, administration of D-ribose leads to an increase of visceral triglycerides in mice (Chen et al., 2019b); and since high levels of D-ribose (Su et al., 2013; Chen et al., 2019a) and high HbA1c (Chen et al., 2017) are associated with diabetes, this raises a possibility that D-ribose could be utilized as a biomarker to assess the therapeutic efficiency.

### Anti-Diabetes Mellitus and Metabolic Syndrome

It is expected that combining APN with treatment against T2DM and other related metabolic disorders might enhance the therapeutic efficiency of targeting of APN. Because of an increased risk of neurodegenerative disorders related to T2DM, previous studies have strongly suggested that treatment of T2DM might be beneficial for and repurposed to therapy of neurodegenerative disorders. Indeed, a recent phase II clinical trial of glucagon-like peptide-1 (GLP-1)/incretin for PD appeared promising (Hölscher, 2012; Athauda et al., 2017), prompting initiation of a subsequent clinical trial of GLP-1/incretin in AD (Gejl et al., 2016). Although metformin has long been used to treat T2DM, there is growing evidence for the benefits of metformin to counteract age-related diseases such as cancer, cardiovascular disease, and neurodegenerative diseases (Rotermund et al., 2018). Moreover, studies are in progress to assess the therapeutic potential of other anti-T2DM molecules, such as DPP-4 inhibitors (Kosaraju et al., 2017) and APN (Sekiyama et al., 2014; Waragai et al., 2018). In relation to the current state of neurodegenerative therapy in relation to T2DM, our current view regarding evolvability may offer several novel insights.

Recently, chronic hypertension has been suggested as one of the largest modifiable risk factors for developing AD (Marfany et al., 2018). Indeed, several epidemiological studies reveal that β-blocker treatment reduces the prevalence of AD in patients suffering from hypertension (Yu et al., 2011). Since it is possible that Aβ evolvability might be promoted not only by amylin protofibrils but also by other molecules, such as cholesterol and catecholamines, it follows that disease-modifying therapies targeted against other metabolic syndrome disorders in addition to T2DM, especially anti-hypertension agents, might increase overall treatment efficacy.

## Concluding Remarks

In the present discussion, the concept of amylin evolvability was shown to provide novel insights into the T2DM and related diseases, which are currently elusive. First, we argue that T1DM and T2DM, etiologically distinct types of DM, might be linked through amylin evolvability. Furthermore, the comorbidity of AD and T2DM in aging might be an antagonistic pleiotropy phenomenon derived from evolvability regulated by AD-relevant APs, including Aβ, tau, and amylin. Importantly, it was suggested that APN resistance might stimulate amylin protofibrils, leading to Aβ protofibrillar formation in evolvability, which might manifest as an increased AD risk driven by T2DM through an antagonistic pleiotropy mechanism in aging. Taken together, a better understanding of the mechanism of evolvability may shed light on novel evolvability-based therapeutic strategies.

## Data Availability Statement

The original contributions presented in the study are included in the article, further inquiries can be directed to the corresponding author.

## Author Contributions

MH conceived the study. MH and GH wrote the article. All authors contributed to the article and approved the submitted version.

## Conflict of Interest

The authors declare that the research was conducted in the absence of any commercial or financial relationships that could be construed as a potential conflict of interest.

The reviewer NK declared a past co-authorship with one of the authors MW to the handling editor.
